# Dryland herbs coordinate leaf protection, absorptive root morphology and lateral-root proliferation along aridity gradients

**DOI:** 10.3389/fpls.2026.1926524

**Published:** 2026-08-20

**Authors:** Jianzhe Ma, Qingshan Xu, Guoan Luo, Jiuxin Lai

**Affiliations:** 1 Anhui Institute of Optics and Fine Mechanics, Hefei Institutes of Physical Science, Chinese Academy of Sciences, Hefei, China; 2 University of Science and Technology of China, Hefei, China; 3 Yuyao Forestry Service Center, Ningbo, China; 4 Zhejiang Academy of Forestry, Hangzhou, China; 5 National Center for Forestry and Grassland Genetic Resources (ZheJiang Academy of Forestry), Hangzhou, China

**Keywords:** aridity gradient, herbaceous plants, leaf traits, leaf–root coordination, root traits

## Abstract

**Introduction:**

Dryland plants must balance atmospheric water demand with below-ground water and nutrient acquisition, yet it remains unclear whether increasing aridity drives coordinated whole-plant trait shifts or instead promotes modular adjustment of leaf and root functions. Here, we examined leaf–root functional coordination in herbaceous plant communities across a continuous aridity gradient in Xinjiang, northwestern China.

**Methods:**

We measured six leaf traits, four root traits and plotlevel environmental variables for 474 matched species-by-plot observations, and combined single-trait models, multivariate trait axes, leaf–root correlation networks, sliding-window coordination analyses and path modeling. Trait responses to aridity were selective rather than uniform. Drier conditions were associated with thicker leaves and higher specific root length, whereas wetter conditions supported greater lateral-root proliferation. By contrast, leaf dry matter content, leaf water content, root length and root diameter showed weak or no direct responses to the aridity gradient. Leaf–root coordination was also trait-specific: leaf size traits were positively linked to root length and root diameter, but negatively associated with specific root length, indicating a separation between below-ground structural support and absorptive efficiency. Environmental models further showed that variation in leaf and root trait axes was associated with aridity through direct and indirect statistical pathways involving soil water availability, microbial carbon processes, SOC–N nutrient status, elevation and life form.

**Results:**

Our results suggest that dryland herbaceous plants do not respond to aridity through a single synchronized whole-plant economic axis. Instead, they maintain function through coupled but modular adjustments involving leaf structural protection, absorptive root morphology and lateral-root proliferation.

**Discussion:**

This modular leaf–root coordination provides a trait-based framework for understanding how herbaceous plants persist along dryland moisture gradients and may improve predictions of plant functional responses to intensifying drought.

## 1 Introduction

Dryland plants operate under a persistent imbalance between atmospheric water demand and below-ground resource supply. Leaves expose plants to light, heat and evaporative demand, whereas roots determine access to spatially and temporally heterogeneous pools of soil water and nutrients. As aridity intensifies, this above- and below-ground balance becomes increasingly important because small changes in water availability can alter plant growth, community composition and ecosystem functioning. Dryland ecosystems can approach ecological thresholds along aridity gradients, where climatic water limitation interacts with soil processes and biotic feedbacks to reorganize ecosystem structure and function (Berdugo et al., 2020, Berdugo et al., 2022). However, the organ-level trait adjustments that precede or accompany these dryland responses remain incompletely understood, particularly for herbaceous plants that complete growth within short and unpredictable windows of soil moisture availability.

Plant functional traits provide a powerful way to examine how dryland species balance water demand and resource acquisition. Leaf traits have received particular attention because they regulate the atmospheric side of plant water balance. Leaf area and leaf dimensions influence light interception, boundary-layer conductance and transpiring surface; leaf thickness reflects tissue construction, internal water storage and resistance to dehydration; leaf dry matter content and leaf water content describe tissue investment and hydration status. Broad-scale trait syntheses have shown that leaf traits capture major axes of plant ecological strategy and provide extensive trait coverage across vegetation types (Kattge et al., 2020; Wang et al., 2022). However, drought responses are not always expressed as uniform shifts in all leaf traits. Recent work in dryland systems suggests that aridity can reorganize trait relationships and separate different functional trade-offs, rather than simply shifting all traits in the same direction (Miao et al., 2025). Thus, the key question is not simply whether leaves respond to aridity, but which components of leaf form and construction are most sensitive to water limitation.

Roots represent the supply side of this whole-plant balance, yet their responses to aridity are often less predictable than those of leaves. Root length can increase soil exploration, root diameter reflects construction cost and transport capacity, specific root length indicates absorptive length per unit biomass, and lateral roots determine local proliferation in resource-rich patches. Recent developments in root ecology have moved away from the idea that below-ground traits follow a single root economics spectrum. Instead, root function is increasingly understood as multidimensional, involving trade-offs among construction cost, absorptive efficiency, architectural deployment and interactions with soil biota (Bergmann et al., 2020; Carmona et al., 2021; Weigelt et al., 2021). Root traits can also explain species distributions along climatic gradients while challenging simple fast–slow expectations of plant economics (Laughlin et al., 2021). This distinction is especially important in drylands. Under severe water limitation, plants may increase absorptive efficiency through high specific root length without necessarily producing larger root systems, whereas under less limiting conditions they may invest in lateral-root proliferation to exploit localized water and nutrient patches. Therefore, drought adaptation may involve shifts among root functional modules rather than a simple increase or decrease in total root size.

A central unresolved issue is whether leaf demand and root supply remain coordinated along continuous aridity gradients. Whole-plant economic theory predicts that traits controlling resource demand and resource acquisition should covary when plant function is integrated across organs. Under this view, aridity should strengthen coordination between leaves and roots because water loss above ground must be matched by water uptake below ground. However, empirical evidence increasingly suggests that above- and below-ground traits are only partially coordinated. Semiarid grassland species can show coordinated leaf, root and seed economics (Mueller et al., 2024), whereas forest communities and desert plant assemblages often show trait-specific or life-form-dependent coordination rather than complete whole-plant integration (Luo et al., 2024; Ma et al., 2024). This creates a conceptual tension between two alternative expectations: aridity may push plants along a single coordinated whole-plant axis, or it may promote modular adjustment in which leaf protection, root absorptive morphology and root proliferation respond in different ways.

Resolving this issue requires field studies that link leaf–root coordination to continuous environmental gradients rather than comparing broad habitat categories alone. Climatic aridity is important, but plants experience drought through local soil water availability, soil nutrient supply, microbial carbon processes and topographic context. Soil water controls cell expansion, root meristem activity and nutrient diffusion. Soil organic carbon and nitrogen influence the resources available for tissue construction. Microbial biomass and carbon-use efficiency reflect microbial processing of organic substrates and may affect nutrient availability and soil carbon retention; microbial carbon-use efficiency has also been identified as an important control of soil carbon storage and dryland carbon cycling (Geyer et al., 2016; Sinsabaugh et al., 2016; Tao et al., 2023; Pei et al., 2024). Elevation can integrate temperature, radiation, hydrological redistribution and soil development. Life form may further modify trait coordination because annual and perennial herbs differ in phenology, lifespan and investment strategy. Consequently, leaf–root coordination in drylands is unlikely to be explained by aridity alone; it may emerge from the joint effects of climate, soil water, below-ground biogeochemistry, topography and plant life history.

Here, we used dryland herbaceous communities across Xinjiang, northwestern China, to test whether aridity reorganizes plant function through a single coordinated whole-plant axis or through modular adjustment of leaf and root functions. We analyzed 474 matched species-by-plot observations, including six leaf traits, four root traits and plot-level climatic, edaphic, microbial, topographic and life-form variables. We focused on three predictions. First, if aridity acts primarily through water-balance constraints, trait responses should be concentrated in traits directly related to leaf exposure, leaf structural protection, absorptive root morphology and root branching, rather than expressed uniformly across all measured traits. Second, if whole-plant coordination is modular rather than complete, leaf size and structural traits should coordinate more strongly with root length and root diameter than with specific root length, reflecting a separation between below-ground structural support and absorptive efficiency. Third, if aridity effects are mediated by local environmental context, leaf–root coordination should be explained not by climatic aridity alone but by pathways involving soil water availability, microbial carbon processing, SOC–N nutrient status, elevation and life form. By testing these predictions, we aimed to identify whether dryland herbs persist along moisture gradients through synchronized whole-plant trait shifts or through coupled but modular reorganization of leaf and root functions.

## 2 Materials and methods

### 2.1 Study area and field sampling

The study was conducted in dryland herbaceous communities across Xinjiang, northwestern China. Xinjiang contains extensive temperate drylands and supports strong regional gradients in precipitation, temperature, elevation and soil water availability, making it suitable for examining plant functional responses to continuous aridity gradients. Sampling sites were distributed across this regional moisture gradient, which was represented by the aridity index (AI). Higher AI values indicate wetter climatic conditions, whereas lower AI values indicate drier conditions.

At each site, herbaceous vegetation was surveyed in 2 m × 2 m quadrats placed in representative patches with no visible recent disturbance. Field sampling was conducted from May to July 2023, during the principal growing season across the study region. The center of each quadrat was recorded using a handheld GPS receiver in the WGS84 coordinate system, and elevation was recorded at the same position. Within each quadrat, herbaceous species were identified and their life forms were recorded as annual, biennial or perennial. For each recorded species, healthy and fully developed individuals were collected for leaf and root trait measurements. Field sampling focused on individuals that showed no visible signs of herbivory, disease or mechanical damage, so that measured traits represented mature functional organs rather than senescent or damaged tissue. For each species in each quadrat, ten healthy, spatially separated individuals were sampled. Individual measurements were averaged to obtain one species-by-plot value for each trait.

The raw field dataset contained 629 records from 87 named plots and 88 coordinate positions. During complete-case matching, 155 records were excluded because at least one required above- or below-ground functional trait was missing; no records were excluded because of missing environmental variables or unsuccessful plot-coordinate matching. After matching plot identity, geographic coordinates, environmental variables, life form and the trait variables required for the main analyses, the final analytical dataset contained 474 matched species-by-plot observations. This dataset was used for all main trait-response, leaf–root coordination, environmental-driver and path analyses. Data hierarchy, variable availability and life-form composition are summarized in **Supplementary Figure 1**.

### 2.2 Climate, topographic and soil variables

Mean annual temperature (MAT), mean annual precipitation (MAP) and monthly climate layers were extracted from WorldClim version 2.1 at 30 arc-second spatial resolution using the plot coordinates (Fick and Hijmans, 2017). Raster extraction was conducted at the coordinate of each plot. MAP was extracted from the annual precipitation layer, and MAT was extracted from the annual mean temperature layer. Potential evapotranspiration (PET) was calculated from WorldClim monthly temperature, solar radiation, wind speed and vapor pressure layers following the FAO-56 Penman–Monteith framework. The aridity index was calculated as:

AI = MAP/PET

Thus, higher AI values indicate wetter climatic conditions and lower AI values indicate greater aridity. MAP was used to validate the climatic moisture gradient, whereas AI was retained as the main climatic predictor in downstream trait models.

Soil samples were collected from the 0–20 cm layer in each quadrat after removing surface litter. Five soil cores were taken within each quadrat and pooled into one composite sample. Fresh soil was stored in sealed bags and transported to the laboratory in a cooled container. One subsample was used immediately for soil water content and microbial analyses, and the remaining soil was air-dried, gently ground and passed through a 2-mm sieve for physicochemical analyses.

Soil water content (SWC) was determined gravimetrically by weighing fresh soil, oven-drying it at 105 °C to constant mass and expressing water loss relative to dry soil mass. Soil pH was measured in a 1:2.5 soil-to-deionized-water suspension using a calibrated pH meter. Soil organic carbon (SOC) was measured using potassium dichromate oxidation with external heating and titration. Total nitrogen (TN) was measured by Kjeldahl digestion followed by distillation and titration. Total phosphorus (TP) was measured after H_2_SO_4_–HClO_4_ digestion using molybdenum–antimony colorimetry on a UV-visible spectrophotometer. SOC and TN were expressed as g kg^+^¹, and TP was expressed as a percentage.

Microbial biomass carbon (MBC) was measured on fresh field-moist soil using chloroform fumigation–extraction (Vance et al., 1987). Paired fumigated and non-fumigated subsamples were extracted with 0.5 mol l^+^¹ K_2_SO_4_ and dissolved organic carbon in the extracts was quantified using a total organic carbon analyzer. MBC was calculated from the difference between fumigated and non-fumigated extracts using an extraction coefficient of 0.45. Microbial carbon use efficiency (CUE) was determined directly from fresh soil by an external analytical laboratory after the samples had been transported under cooled conditions. The authors did not conduct a substrate-addition incubation; therefore, incubation duration, incubation temperature and carbon source are not applicable to this dataset. CUE values were reported by the analytical laboratory and were used in the statistical analyses without recalculation. The laboratory report did not provide the underlying growth and respiration measurements or a calculation equation; consequently, these method-specific details were unavailable for retrospective reporting.

### 2.3 Leaf and root trait measurements

Leaf traits were measured on mature, fully expanded and undamaged leaves collected from the sampled individuals. Leaf thickness was measured with a digital caliper at three positions on each leaf while avoiding the midrib, and the mean value was used for analysis. Leaf area was measured with an LI-3100C leaf area meter (LI-COR Biosciences, Lincoln, NE, USA). Leaf length and leaf width were measured with a digital caliper or from scanned leaf images. Fresh leaf mass was recorded immediately after sampling. Leaves were then oven-dried at 65 °C for 48 h to constant mass and weighed again. Leaf dry matter content (LDMC) was calculated as dry mass per unit fresh mass, and leaf water content was calculated from the difference between fresh and dry mass.

Root systems were excavated from the same individuals used for leaf sampling whenever possible. Soil particles were removed by gentle washing over a fine sieve to minimize root loss. Clean roots were spread in water to minimize overlap and scanned at 400 dpi using an Epson Perfection V800 scanner (Seiko Epson Corporation, Japan). Total root length and mean root diameter were obtained from the scanned images using WinRHIZO image-analysis software (Regent Instruments, Canada). Lateral root number was counted from cleaned root images after separating lateral branches from the main root axis. Roots were then oven-dried at 65 °C for 48 h to constant mass. Specific root length was calculated as total root length per unit dry root mass and expressed as m g^+^¹.

The measured leaf traits were leaf thickness, leaf dry matter content, leaf water content, leaf area, leaf length and leaf width. The measured root traits were root length, root diameter, lateral root number and specific root length. Together, these traits captured leaf structural investment, leaf size, leaf hydration, root size, absorptive morphology and root branching.

### 2.4 Data analysis

All analyses were conducted on the matched species-by-plot dataset. Plot-level climate, soil, microbial and topographic variables were linked to each species trait record by plot identity and geographic coordinates. Life form was linked to each species-by-plot observation and used as a categorical biological predictor in downstream models. Trait variables with strongly right-skewed distributions were transformed before analysis. Leaf area, lateral root number and specific root length were log_10_-transformed after adding one to allow zero values. Continuous environmental and trait variables were standardized before multivariate analyses and standardized-effect models, allowing regression coefficients to be compared among variables measured on different scales.

**Figure 1:**
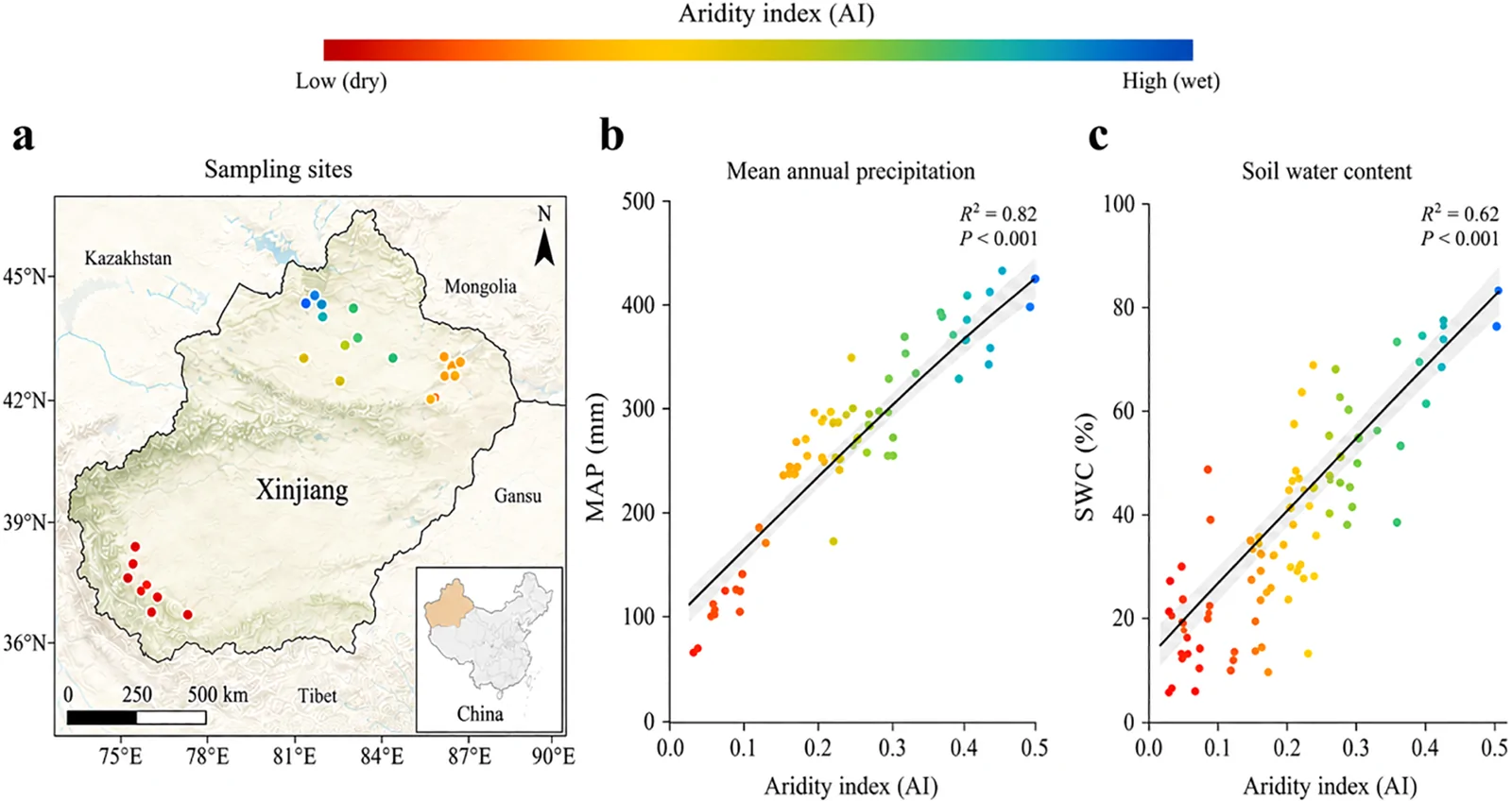
Sampling sites and the aridity gradient across Xinjiang. **(a)** Locations of the sampling sites across Xinjiang, northwestern China, mapped in the WGS84 geographic coordinate system (EPSG:4326) and displayed in a Plate Carrée (equirectangular) layout. The panel includes a geographic graticule, scale bar, north arrow and national-location inset. Points are colored by aridity index (AI), with lower AI values indicating drier conditions and higher values indicating wetter conditions. **(b)** Relationship between AI and mean annual precipitation (MAP). **(c)** Relationship between AI and soil water content (SWC). Lines indicate fitted linear regressions with 95% confidence intervals. This figure shows that the sampling sites covered a continuous climate–soil moisture gradient.

The aridity gradient was first evaluated at the plot or coordinate level. Sampling points were mapped using the WGS84 geographic coordinate system (EPSG:4326) and displayed in a Plate Carrée (equirectangular) layout, with a geographic graticule, scale bar, north arrow and national-location inset, and linear relationships between AI, MAP and SWC were fitted to test whether the WorldClim-derived climatic moisture gradient corresponded to local soil moisture. MAP was used to validate the climatic gradient, whereas AI was retained as the main climatic predictor in downstream trait models. These analyses generated the gradient validation shown in **Figure 1**. Subsequent single-trait, multivariate, coordination, environmental-driver and path analyses used the 474 matched species-by-plot observations.

Composite trait and environmental axes were constructed using principal component analysis (PCA) on centered and scaled variables. The leaf trait axis was derived from leaf thickness, leaf dry matter content, leaf water content, log-transformed leaf area, leaf length and leaf width. The root trait axis was derived from root length, root diameter, log-transformed lateral root number and log-transformed specific root length. For environmental predictors, microbial biomass carbon and carbon use efficiency were summarized as a microbial carbon axis, whereas soil organic carbon, total nitrogen and total phosphorus were summarized as a SOC–N nutrient axis. The first principal component of each group was used in downstream models to reduce dimensionality and to represent dominant covariation among related variables. PCA loadings were examined to support ecological interpretation of each axis. The predictor correlation matrix, VIF values before and after variable reduction, and PCA loadings are shown in **Supplementary Figure 3**.

Predictor redundancy was assessed before fitting environmental models. Spearman correlations were used to describe pairwise associations among candidate environmental predictors, and variance inflation factors were used to evaluate multicollinearity. MAP was retained for gradient validation but was not included together with AI in the final multivariable models because both variables represented climatic moisture availability. The final predictor set consisted of AI, SWC, microbial carbon axis, SOC–N nutrient axis, elevation and life form. Life form was coded as a perennial contrast, with non-perennial herbs as the reference group. Sensitivity analyses were performed for the main annual and perennial life-form subsets to examine whether AI–trait relationships were broadly consistent across life forms; panel-specific sample sizes reflected the observations available for each trait group, and these results are shown in **Supplementary Figure 2**.

**Figure 2:**
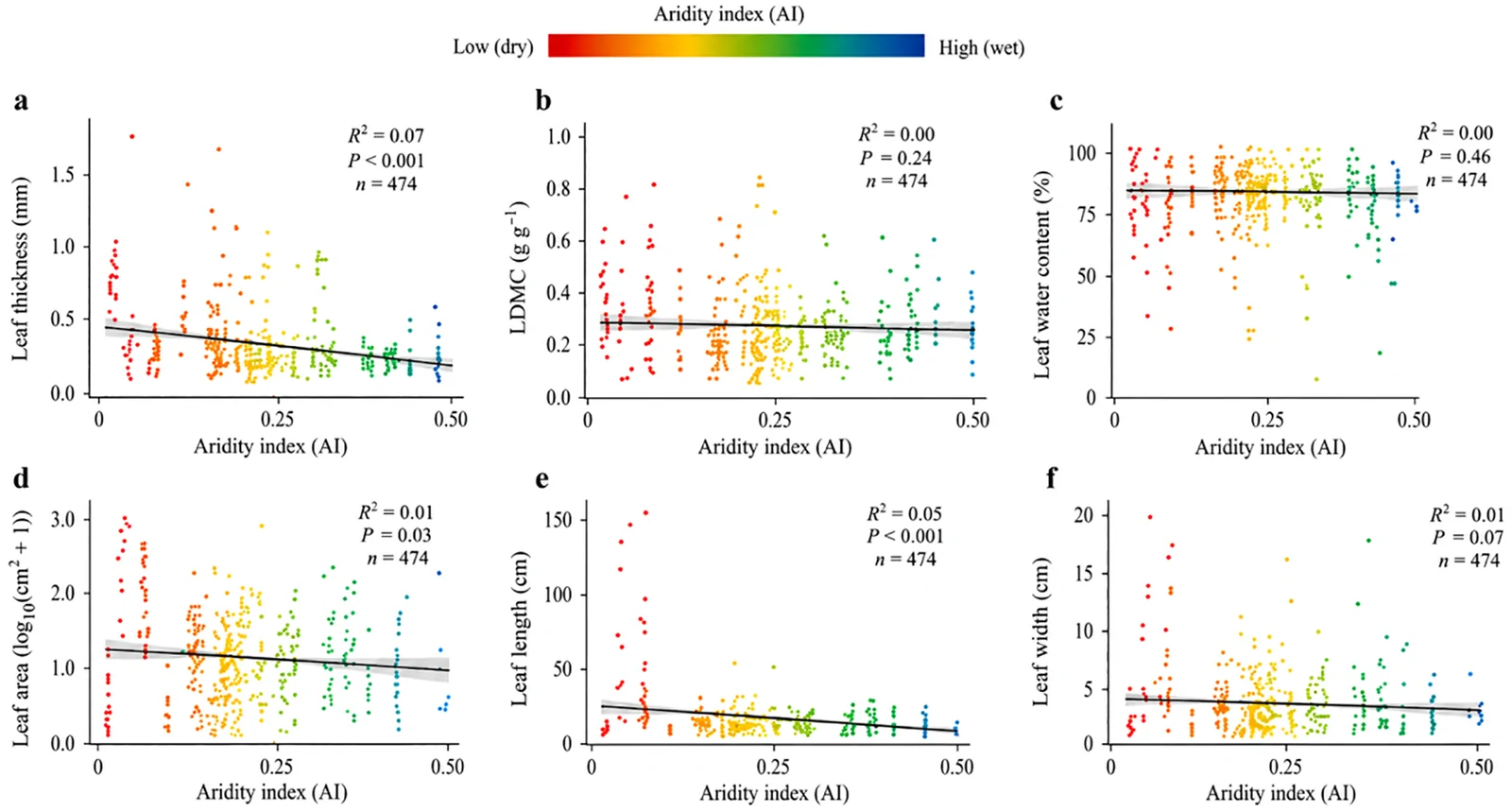
Leaf trait responses along the aridity gradient. Relationships between aridity index (AI) and six leaf functional traits across 474 matched species-by-plot observations. **(a)** Leaf thickness. **(b)** Leaf dry matter content (LDMC). **(c)** Leaf water content. **(d)** Leaf area, log-transformed as log10(cm² + 1). **(e)** Leaf length. **(f)** Leaf width. Points are colored by AI. Lines indicate fitted linear relationships with 95% confidence intervals. R², P values and sample sizes are shown in each panel.

**Figure 3:**
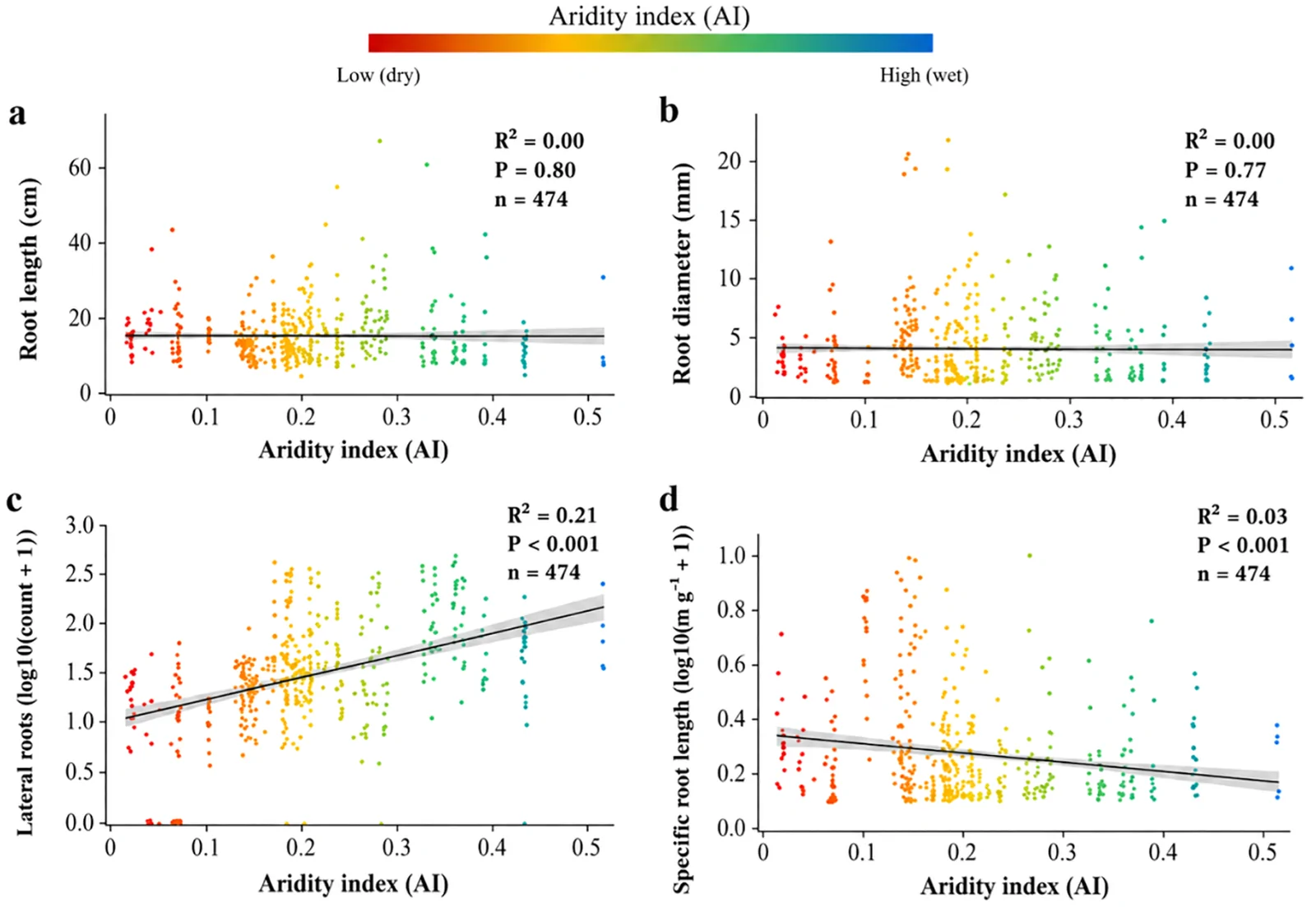
Root trait responses along the aridity gradient. Relationships between aridity index (AI) and four root functional traits across 474 matched species-by-plot observations. **(a)** Root length. **(b)** Root diameter. **(c)** Lateral root number, log-transformed as log10(count + 1). **(d)** Specific root length, log-transformed as log10(m g^+^¹ + 1). Points are colored by AI. Lines indicate fitted linear relationships with 95% confidence intervals. R², P values and sample sizes are shown in each panel.

Single-trait responses to aridity were analyzed with AI as a continuous predictor. Separate models were fitted for the six leaf traits and four root traits. For leaf traits, the response variables were leaf thickness, leaf dry matter content, leaf water content, log-transformed leaf area, leaf length and leaf width. For root traits, the response variables were root length, root diameter, log-transformed lateral root number and log-transformed specific root length. These models were used to identify which individual traits changed along the continuous moisture gradient before testing multivariate trait axes and leaf–root coordination. Fitted relationships, coefficients of determination, P values and sample sizes are reported in **Figures 2**, **3**. Because higher AI indicates wetter conditions and lower AI indicates drier conditions, all AI–trait relationships were interpreted in relation to this direction of the moisture gradient. The ten trait-level models represented *a priori*, trait-specific hypotheses rather than an exploratory screening exercise; therefore, unadjusted two-sided P values were retained. To support interpretation beyond statistical significance, effect directions, coefficients of determination, exact P values and sample sizes are reported for every trait.

**Figure 4:**
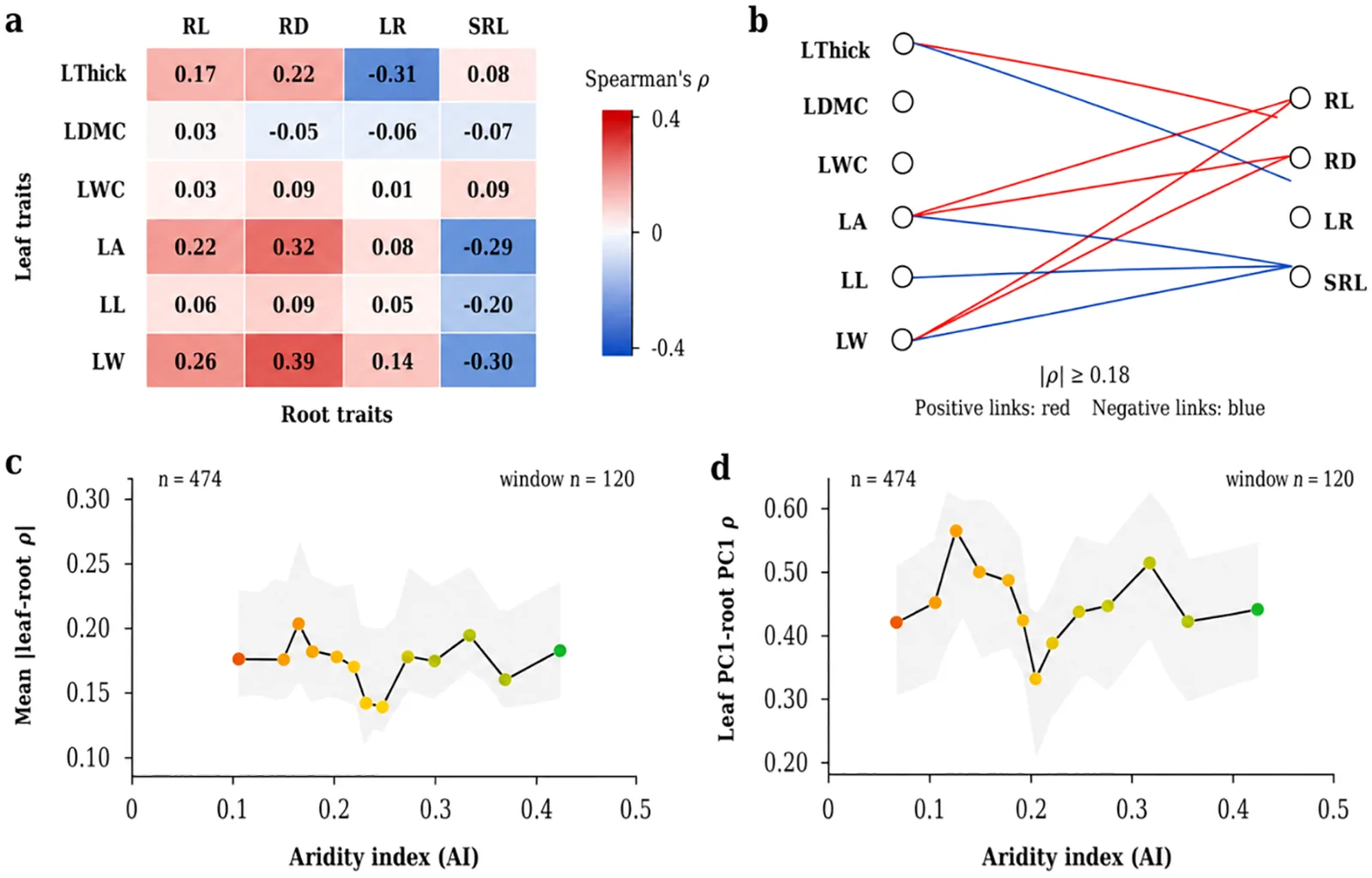
Leaf–root trait coordination along the aridity gradient. **(a)** Spearman correlation matrix between leaf traits and root traits. Red indicates positive correlations and blue indicates negative correlations. **(b)** Leaf–root trait network based on correlations with |ρ| ≥ 0.18. Red links indicate positive correlations and blue links indicate negative correlations. **(c)** Sliding-window changes in mean absolute leaf–root correlation strength along the AI gradient. **(d)** Sliding-window correlation between leaf PC1 and root PC1 along the AI gradient. Sliding-window analyses were based on 474 observations with a window size of 120 observations. Shaded areas indicate uncertainty around window-based estimates.

Leaf–root coordination was quantified in three complementary ways. First, pairwise Spearman correlations were calculated between all leaf traits and all root traits to identify positive and negative associations between above-ground and below-ground functional traits. Spearman correlations were used because they are robust to non-normal distributions and monotonic but non-linear relationships. Second, a leaf–root trait network was constructed from the pairwise correlation matrix. Leaf and root traits were represented as nodes, and trait associations were represented as edges. To retain the stronger associations while avoiding a visually saturated network, only correlations with absolute Spearman correlation coefficients of |ρ| ≥ 0.18 were shown. Positive and negative links were displayed separately to distinguish coordinated increases from trade-offs between leaf and root traits. The network was used as a visualization of the dominant leaf–root association structure rather than as a causal model. Third, observations were ordered by AI and analyzed using a sliding-window approach with a window size of 120 observations. Within each window, two coordination metrics were calculated: the mean absolute leaf–root correlation across all pairwise leaf–root trait combinations, and the correlation between leaf PC1 and root PC1. The first metric represented overall leaf–root association strength, whereas the second represented coordination between dominant multivariate leaf and root trait axes. These sliding-window analyses evaluated whether leaf–root coordination changed along the aridity gradient rather than assuming a single correlation structure for the entire dataset. The sliding-window statistics were used only to describe changes along the AI gradient in **Figures 4c, d** and were not entered as species-by-plot responses in the mixed or path models. For those models, an observation-level coordination index was calculated separately for each species-by-plot record as CI_i_ = −|z(leaf PC1_i_) − z(root PC1_i_)|, where the two PC1 scores were standardized across the 474 observations. Higher values (values closer to zero before final standardization) indicate closer alignment between the dominant leaf and root trait axes. The resulting 474 CI values were standardized before the environmental-driver and path analyses.

Environmental driver models were fitted to evaluate how climate, soil water, microbial carbon processes, soil carbon–nutrient status, elevation and life form were associated with multivariate trait structure and leaf–root coordination. Standardized linear mixed-effects models were fitted separately for the leaf trait axis, root trait axis and leaf–root coordination index. The fixed effects were AI, SWC, microbial carbon axis, SOC–N nutrient axis, elevation and life form. Plot identity was included as a random intercept because species measured within the same quadrat shared the same environmental exposure and sampling context. Species identity was not included as a second random effect because the analysis targeted community-wide trait variation, including species turnover along the gradient, whereas plot was the primary sampling cluster for the plot-level environmental predictors. Accordingly, the model accounts for dependence among species within plots but does not fully partition dependence arising when the same species occurs in different plots. Standardized coefficients, 95% confidence intervals, P values and marginal R² values were extracted for comparison among predictors. Marginal R² represented the proportion of variance explained by fixed effects. Because AI and SWC represent related but distinct components of water availability, their coefficients were interpreted as conditional effects when included in the same model: AI represented the broad climatic moisture gradient, whereas SWC represented local soil water availability at the sampling quadrat.

Path analysis was used to evaluate whether the hypothesized environmental–trait structure was consistent with the observed covariance among aridity, local soil conditions, trait axes and leaf–root coordination. The model followed the structure of the measurement system and the proposed ecological pathway: AI and elevation were linked to SWC and soil biogeochemical axes; SWC, microbial carbon axis and SOC–N nutrient axis were linked to leaf and root trait axes; and leaf and root trait axes were linked to leaf–root coordination. All variables were standardized before path analysis. Direct, indirect and total effects were extracted from the fitted path model, together with the explained variance of SWC, microbial carbon axis, SOC–N nutrient axis, leaf trait axis, root trait axis and leaf–root coordination. The path model was used to summarize hypothesized environmental–trait pathways and was interpreted as observational evidence for structured associations rather than as experimental proof of causality.

Model residuals, normal Q–Q patterns, random-effect distributions and Cook’s distance were examined for the main model classes. These diagnostics were used to evaluate residual structure, approximate normality, random-effect behavior and influential observations. Diagnostic plots are shown in **Supplementary Figure 4**. All statistical analyses were conducted in R. Raster climate extraction was performed using spatial analysis packages in R. PCA was conducted on centered and scaled variables. VIFs were calculated using the car package. Linear mixed-effects models were fitted and diagnosed using lme4 and related diagnostic tools. Path models were fitted using piecewise SEM. Marginal R² for mixed models followed the fixed-effect variance definition of Nakagawa and Schielzeth.

## 3 Results

The sampled sites captured a clear climate–soil moisture gradient across Xinjiang (**Figure 1**). Sampling locations were distributed across a broad range of aridity index values, with lower AI indicating drier conditions and higher AI indicating wetter conditions (**Figure 1a**). AI was positively related to mean annual precipitation and soil water content, confirming that the WorldClim-derived climatic moisture gradient was reflected in both regional precipitation and local soil moisture conditions (**Figures 1b, c**). This gradient provided the environmental basis for testing how herbaceous leaf traits, root traits and leaf–root coordination changed along continuous variation in water availability.

Trait responses to the aridity gradient were selective rather than uniform (**Figures 2**, **3**). Among leaf traits, leaf thickness showed the clearest response to AI, decreasing significantly towards wetter conditions (R² = 0.07, *P* < 0.001, n = 474; **Figure 2a**). Leaf area and leaf length also decreased with increasing AI, but these relationships were weaker, especially for leaf area (leaf area: R² = 0.01, *P* = 0.03; leaf length: R² = 0.05, *P* < 0.001; **Figures 2d, e**). By contrast, leaf dry matter content, leaf water content and leaf width showed no significant response to AI (LDMC: R² = 0.00, *P* = 0.24; leaf water content: R² = 0.00, *P* = 0.46; leaf width: R² = 0.01, *P* = 0.07; **Figures 2b, c, f**). Thus, leaf adjustment along the moisture gradient was concentrated mainly in leaf thickness and, to a lesser extent, leaf size dimensions rather than in bulk tissue dry matter or water content.

Root traits showed a different pattern of response (**Figure 3**). Root length and root diameter were unrelated to AI (root length: R² = 0.00, *P* = 0.80; root diameter: R² = 0.00, *P* = 0.77; **Figures 3a, b**), indicating that the aridity gradient did not primarily alter overall root size or mean root thickness. In contrast, lateral root number increased strongly towards wetter conditions after log transformation (R² = 0.21, *P* < 0.001; **Figure 3c**), whereas specific root length decreased with increasing AI (R² = 0.03, *P* < 0.001; **Figure 3d**). Therefore, the clearest below-ground responses were expressed through root branching and absorptive morphology rather than through total root length or root diameter. Together with the leaf results, these patterns indicate that dryland herbs did not shift all measured traits in a single direction along the aridity gradient; instead, responses were concentrated in a small set of leaf structural, root absorptive and root branching traits.

Leaf–root coordination was trait-specific rather than uniform across all trait combinations (**Figure 4**). Pairwise correlations showed that leaf size-related traits were generally positively associated with root structural traits (**Figure 4a**). The strongest positive correlations were observed between leaf width and root diameter (Spearman’s ρ = 0.39), leaf area and root diameter (ρ = 0.32), leaf width and root length (ρ = 0.26), leaf area and root length (ρ = 0.22), and leaf thickness and root diameter (ρ = 0.22). In contrast, several leaf traits were negatively associated with absorptive root morphology or branching. The strongest negative correlations were leaf thickness with lateral roots (ρ = −0.31), leaf width with specific root length (ρ = −0.30), leaf area with specific root length (ρ = −0.29), and leaf length with specific root length (ρ = −0.20). The leaf–root network based on |ρ| ≥ 0.18 retained these dominant positive links between leaf size and root length or diameter, as well as negative links between leaf size traits and specific root length (**Figure 4b**). Sliding-window analyses further showed that coordination changed along the AI gradient: mean absolute leaf–root correlations ranged approximately from 0.14 to 0.20, whereas the correlation between leaf PC1 and root PC1 ranged from about 0.33 to 0.56 (**Figures 4c, d**). These results indicate partial coordination rather than complete synchronization between leaves and roots, with a structural coordination axis linking larger leaves to longer or thicker roots and an absorptive axis separating leaf size from high specific root length.

**Figure 5:**
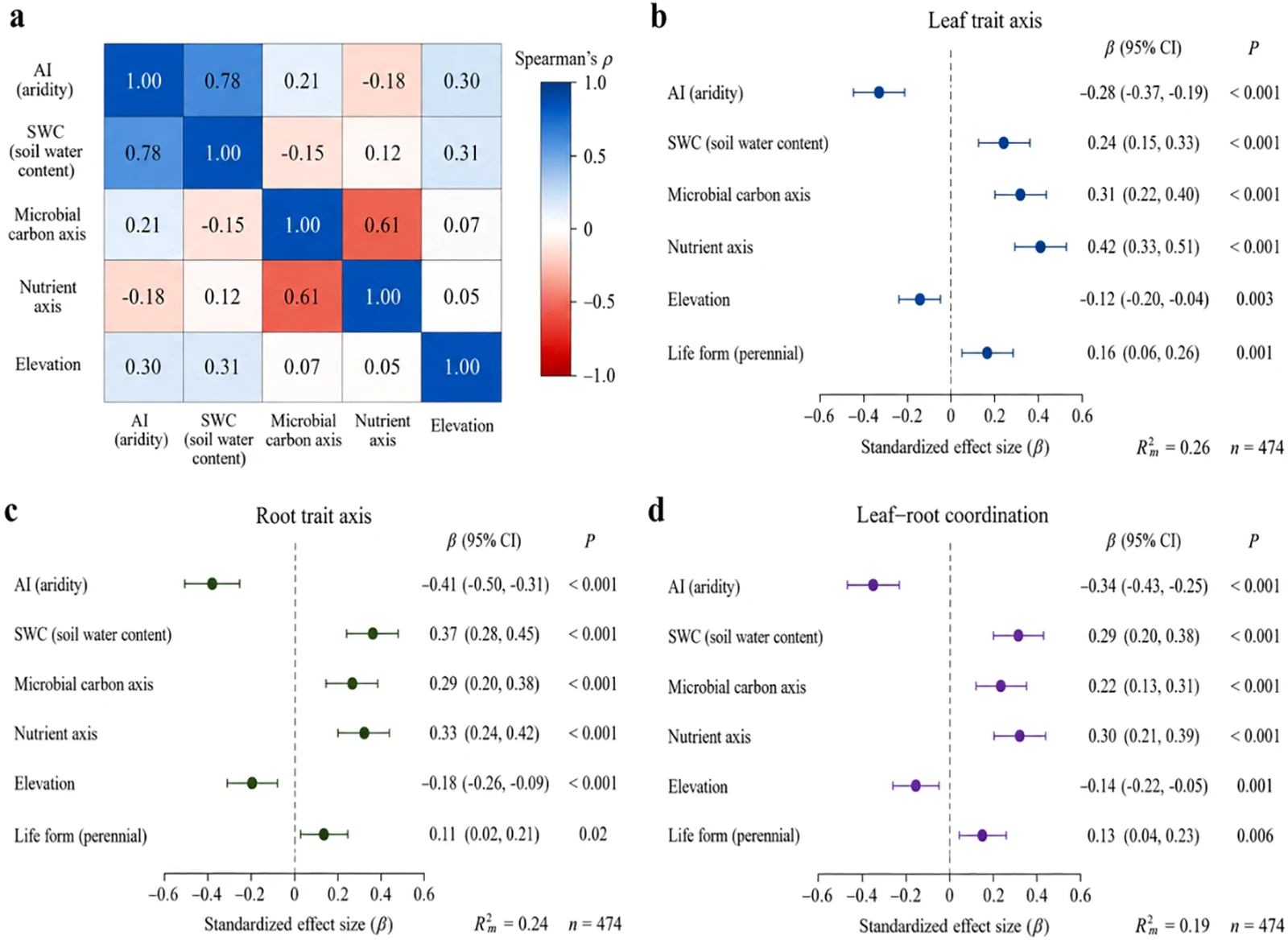
Environmental controls on leaf traits, root traits and leaf–root coordination. **(a)** Spearman correlation matrix among retained environmental predictors, including aridity index (AI), soil water content (SWC), microbial carbon axis, nutrient axis and elevation. **(b–d)** Standardized effects of environmental predictors on the leaf trait axis **(b)**, root trait axis **(c)** and leaf–root coordination **(d)**. Points indicate standardized regression coefficients and horizontal bars indicate 95% confidence intervals. Positive coefficients indicate positive associations with the corresponding response axis, whereas negative coefficients indicate negative associations. Marginal R² values and sample sizes are shown in each panel.

Environmental driver models showed that multivariate leaf and root trait structure was associated with both climatic aridity and local environmental context (**Figure 5**). The leaf trait axis was negatively associated with AI (β = −0.28, 95% CI = −0.37 to −0.19, *P* < 0.001) and elevation (β = −0.12, 95% CI = −0.20 to −0.04, *P* = 0.003), but positively associated with soil water content (β = 0.24, 95% CI = 0.15 to 0.33, *P* < 0.001), microbial carbon axis (β = 0.31, 95% CI = 0.22 to 0.40, *P* < 0.001), SOC–N nutrient axis (β = 0.42, 95% CI = 0.33 to 0.51, *P* < 0.001) and perennial life form (β = 0.16, 95% CI = 0.06 to 0.26, *P* = 0.001; marginal R² = 0.26; **Figure 5b**). The root trait axis showed a similar structure, with negative associations with AI (β = −0.41, 95% CI = −0.50 to −0.31, *P* < 0.001) and elevation (β = −0.18, 95% CI = −0.26 to −0.09, *P* < 0.001), and positive associations with soil water content (β = 0.37, 95% CI = 0.28 to 0.45, *P* < 0.001), microbial carbon axis (β = 0.29, 95% CI = 0.20 to 0.38, *P* < 0.001), SOC–N nutrient axis (β = 0.33, 95% CI = 0.24 to 0.42, *P* < 0.001) and perennial life form (β = 0.11, 95% CI = 0.02 to 0.21, *P* = 0.02; marginal R² = 0.24; **Figure 5c**). Leaf–root coordination was also negatively associated with AI and elevation, but positively associated with soil water content, microbial carbon axis, SOC–N nutrient axis and perennial life form; this model explained 19% of variation in leaf–root coordination (**Figure 5d**). Because AI and SWC were included simultaneously, these coefficients represent conditional effects: AI captured the broader climatic moisture gradient, whereas SWC represented local soil water availability at the sampling quadrat.

**Figure 6:**
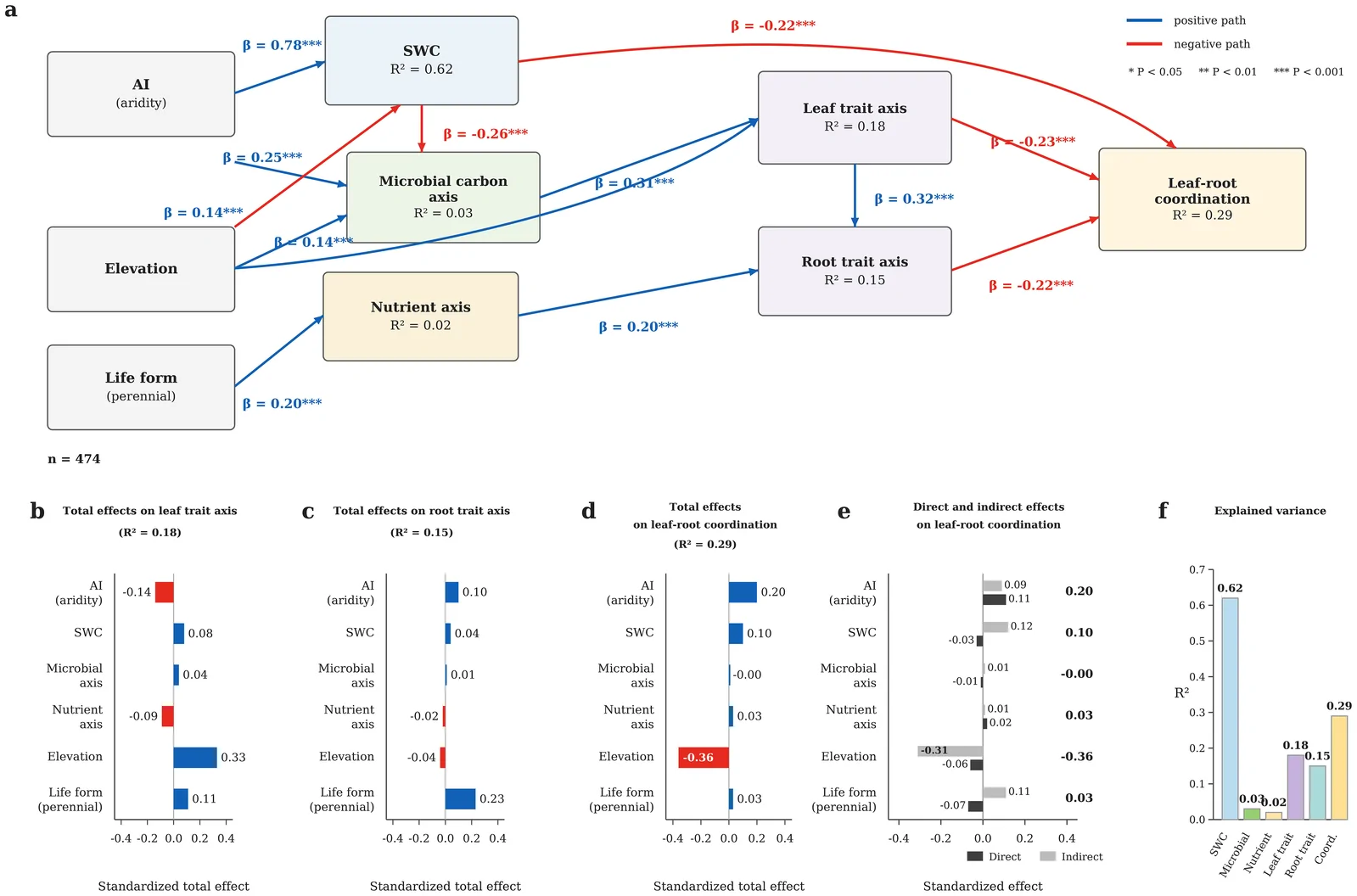
Pathways linking aridity, environmental factors, trait axes and leaf–root coordination. **(a)** Path model showing direct relationships among aridity index (AI), soil water content (SWC), microbial carbon axis, nutrient axis, elevation, life form, leaf trait axis, root trait axis and leaf–root coordination. Blue arrows indicate positive paths and red arrows indicate negative paths. Numbers on arrows are standardized path coefficients; asterisks indicate significance levels. Values inside boxes indicate explained variance. **(b–d)** Total effects of predictors on the leaf trait axis **(b)**, root trait axis **(c)** and leaf–root coordination **(d, e)** Direct and indirect effects on leaf–root coordination. **(f)** Explained variance of variables included in the path model. The analysis was based on 474 matched species-by-plot observations.

The path model was consistent with a hierarchical environmental–trait structure linking aridity, soil water, biogeochemical axes, trait axes and leaf–root coordination (**Figure 6**). AI was strongly and positively linked to soil water content (β = 0.78) and was also positively linked to the microbial carbon axis (β = 0.25; **Figure 6a**). Elevation was positively linked to both soil water content and the microbial carbon axis, whereas soil water content was negatively linked to the microbial carbon axis (**Figure 6a**). Downstream, the microbial carbon axis was positively linked to the leaf trait axis, the SOC–N nutrient axis was positively linked to the root trait axis, and the root trait axis was positively linked to the leaf trait axis (**Figure 6a**). Both leaf and root trait axes were negatively linked to the leaf–root coordination index, reflecting the orientation of the PCA axes and the definition of the coordination metric rather than an absence of leaf–root association (**Figure 6a**). Soil water content also showed a negative direct path to leaf–root coordination in the path model (β = −0.22), whereas its standardized total effect was positive (0.10), indicating that direct and indirect components had opposite signs (**Figures 6a, d, e**). The model explained 62% of variation in soil water content, 3% in microbial carbon axis, 2% in SOC–N nutrient axis, 18% in the leaf trait axis, 15% in the root trait axis and 29% in leaf–root coordination (**Figures 6a, f**). Total-effect estimates indicated that elevation had the strongest association with leaf–root coordination, followed by AI and soil water content (**Figures 6d, e**). Overall, the path analysis supported the view that leaf–root coordination was associated not with aridity alone, but with linked climatic, topographic, soil-water and biogeochemical gradients.

## 4 Discussion

Our study shows that dryland herbaceous plants along the Xinjiang aridity gradient did not respond to water limitation through a uniform shift of all measured leaf and root traits. Instead, the dominant pattern was a modular reorganization of above- and below-ground functions. This finding extends the whole-plant economics perspective proposed by Wright et al. (2004); Reich (2014) and Díaz et al. (2016), but also supports the view that plant ecological strategies cannot be reduced to a single axis when below-ground traits are considered. Recent global syntheses have similarly shown that plant form and function are multidimensional, especially when fine-root traits are integrated with above-ground traits (Kattge et al., 2020; Carmona et al., 2021; Wang et al., 2022). In our study, drier conditions were associated with thicker leaves and higher specific root length, whereas wetter conditions supported greater lateral-root proliferation. Leaf–root coordination was also selective: leaf size-related traits were positively associated with root length and root diameter but negatively associated with specific root length. These results indicate that dryland herbs are neither fully synchronized whole-plant phenotypes nor collections of independent organs. Rather, they maintain function through coupled but partially decoupled modules involving leaf structural protection, root absorptive morphology and local root proliferation.

The selective nature of leaf responses is important because it indicates that aridity did not impose a generalized conservative leaf syndrome. Leaf thickness increased towards the drier end of the gradient, whereas leaf dry matter content and leaf water content were not significantly related to AI. Trait handbooks such as Pérez-Harguindeguy et al. (2013) emphasize that leaf thickness, LDMC and leaf water content represent related but non-identical aspects of leaf construction and hydration. Therefore, the increase in leaf thickness without corresponding shifts in LDMC or leaf water content suggests that the dominant leaf adjustment was structural rather than a broad change in tissue density or bulk hydration. Thicker leaves may provide greater buffering against rapid dehydration, high irradiance and thermal stress, but the weak responses of leaf area and leaf length caution against interpreting the pattern as a simple shift from acquisitive to conservative strategies. This agrees with Kattge et al. (2020) and Wang et al. (2022), who showed that leaf traits capture multiple functional dimensions across broad vegetation types. It also aligns with the dryland leaf-trait network study of Miao et al. (2025), which suggested that aridity can reorganize trait relationships rather than shift all leaf traits in the same direction.

Root responses followed a different but functionally coherent pattern. Total root length and root diameter were not significantly related to AI, whereas specific root length was higher at the drier end and lateral root number increased towards wetter conditions. This pattern supports recent root-trait theory, especially the multidimensional root economics framework proposed by Bergmann et al. (2020) and the below-ground form–function framework of Weigelt et al. (2021). Rather than producing larger or thicker root systems under drought, dryland herbs appeared to shift between two root modules: high absorptive length per unit biomass under drier conditions and greater branching proliferation under wetter conditions. Kong et al. (2019) showed that root trait relationships can be nonlinear, and Laughlin et al. (2021) further demonstrated that root traits can explain species distributions along climatic gradients while challenging simple fast–slow expectations. Our results are consistent with this perspective because the strongest below-ground responses were expressed through specific root length and lateral-root number, not through total root size.

The contrast between high specific root length under drier conditions and greater lateral-root number under wetter conditions provides a mechanistic interpretation of dryland herb strategies. Freschet et al. (2021a) emphasized the importance of distinguishing root architecture, morphology and function, and our results support that distinction. In highly water-limited environments, increasing absorptive length per unit biomass may be a lower-cost strategy than expanding total root size or producing many lateral branches. This strategy may improve contact with scarce or transient water pulses without requiring large biomass investment. Under relatively wetter conditions, however, water availability may relax constraints on meristem activity and carbon allocation, allowing plants to increase lateral branching and exploit localized resource patches. Freschet et al. (2021b) argued that root traits influence plant and ecosystem functioning through multiple pathways, and our data suggest that absorptive efficiency and branching proliferation represent distinct below-ground responses along the dryland moisture gradient.

The leaf–root correlation structure further supports a modular coordination framework. Leaf width and leaf area were positively associated with root diameter and root length, whereas leaf size traits were negatively associated with specific root length. Whole-plant economic theory predicts that resource demand and acquisition should be coordinated across organs, but our results show that this coordination depends strongly on which traits are compared. Mueller et al. (2024) reported coordinated leaf, root and seed economics in semiarid grasslands, whereas Luo et al. (2024) found only partial leaf–root coordination in subtropical forest communities. Ma et al. (2024) further showed that leaf–root coordination differs among herbaceous and shrub species in desert ecosystems. Our results refine these findings by separating a structural coordination module from an absorptive module. Larger leaves may require longer or thicker roots for transport, anchorage or hydraulic support, whereas high specific root length represents absorptive efficiency per unit biomass rather than structural investment. Therefore, the negative association between leaf size and specific root length suggests a trade-off between above-ground display and below-ground absorptive efficiency.

The sliding-window analyses indicate that leaf–root coordination was not fixed across the entire aridity gradient. Instead, both mean absolute leaf–root correlation strength and the correlation between leaf PC1 and root PC1 varied along AI. This result helps explain why previous studies have reported inconsistent degrees of above- and below-ground coordination across ecosystems. As Carmona et al. (2021) showed, fine-root traits occupy a broad functional space that does not always map directly onto above-ground trait spectra. Similarly, Laughlin et al. (2021) argued that climatic gradients can reveal root trait trade-offs that depart from simple economic expectations. Our sliding-window results suggest that coordination itself is environmentally contingent. In drylands, the coupling between leaf demand and root supply may strengthen or weaken depending on local soil water, nutrient status, microbial carbon processes and topographic context.

Environmental driver models showed that trait axes and leaf–root coordination were associated not only with climatic aridity but also with local soil water, microbial carbon processes, SOC–N status, elevation and life form. Berdugo et al. (2020) demonstrated that dryland ecosystems can approach functional thresholds along aridity gradients, and Berdugo et al. (2022) further emphasized that such thresholds emerge through interactions among climate, soil processes and biotic feedbacks. Our results are consistent with this framework. AI captured the broad climatic moisture gradient, whereas SWC represented local soil water availability at the quadrat scale. The opposite conditional signs of AI and SWC in multivariable models should therefore not be viewed as contradictory. Rather, they indicate that regional climatic aridity and local soil moisture describe different components of the dryland water environment. Climate defines the broad template, but local soil water represents the realized moisture conditions available to roots.

The positive associations of microbial carbon and SOC–N axes with trait structure suggest that dryland leaf–root coordination is embedded in below-ground biogeochemical context. Geyer et al. (2016) and Sinsabaugh et al. (2016) highlighted microbial carbon use efficiency as a key process regulating the fate of metabolized organic carbon. More recently, Tao et al. (2023) showed that microbial carbon use efficiency can promote global soil carbon storage, whereas Pei et al. (2024) linked aridity-driven changes in microbial carbon use efficiency to soil carbon dynamics. In our study, the microbial carbon axis was positively associated with the leaf trait axis, suggesting that above-ground trait expression covaried with microbial carbon processing. The SOC–N nutrient axis was positively associated with the root trait axis, consistent with the idea that organic carbon and nitrogen availability shape root construction, branching and absorptive morphology. These associations do not prove direct plant–microbe causality, but they indicate that climatic aridity alone is insufficient to explain leaf–root trait organization in dryland herbaceous communities.

Elevation had a strong total association with leaf–root coordination in the path model, suggesting that topographic context integrated multiple environmental constraints. In drylands, elevation can influence temperature, radiation, growing-season length, runoff redistribution, soil development and vegetation composition. Therefore, the elevation effect should not be interpreted as a single causal mechanism. Instead, it likely reflects the combined influence of several landscape-level processes on plant water demand and below-ground resource supply. This interpretation is consistent with the dryland threshold framework of Berdugo et al. (2022), in which climatic effects are amplified or buffered by local soil and landscape processes. Life form also contributed to variation in multivariate trait structure and coordination. Annual and perennial herbs differ in phenology, lifespan and allocation strategy, and Ma et al. (2024) showed that life form can modify leaf–root coordination in desert plants. The positive association of perennial life form with trait axes and coordination suggests that lifespan strategy modulates how herbs balance leaf demand and root supply.

The path model provided an integrated view of these environmental and trait relationships. AI was strongly linked to soil water content, confirming that the climatic gradient was transmitted to local moisture conditions. Downstream, microbial carbon and SOC–N axes were associated with leaf and root trait axes, and the root trait axis was linked to the leaf trait axis. Piecewise structural equation modeling, as described by Lefcheck (2016), is useful for evaluating hypothesized ecological pathways in complex observational datasets. However, the path model should be interpreted as a hypothesized covariance structure rather than experimental evidence of causality. Nakagawa and Schielzeth (2013) also emphasized the importance of variance partitioning in mixed models, and in our case the explained variance values indicate that soil water and leaf–root coordination were better captured than microbial or nutrient axes. The negative paths from leaf and root trait axes to the coordination index likely reflect PCA-axis orientation and the definition of the coordination metric, rather than a biological absence of coordination.

Several limitations should be acknowledged. First, individual trait responses to AI were often modest, especially for leaf area and specific root length. Therefore, the main conclusion is not that aridity strongly shifted every trait, but that a limited set of traits showed functionally coherent responses that together revealed modular adjustment. Second, the dataset was based on field observations along a natural gradient, so unmeasured environmental variables, species turnover and local site history may also contribute to the observed patterns. Third, leaf and root traits were measured at the species-by-plot level, and coordination was inferred from trait correlations and multivariate axes rather than direct measurements of whole-plant hydraulic or carbon fluxes. Rodriguez-Alarcon et al. (2024) showed that intraspecific variation in fine-root traits can be substantial in herbaceous species under drought, indicating that future studies should further separate intraspecific plasticity from species turnover. Combining trait measurements with physiological rates, fine-root dynamics, isotopic water uptake, mycorrhizal associations and experimental drought manipulation would allow the mechanisms proposed here to be tested more directly. Fourth, because species identity was not included as a crossed random effect, the environmental models do not fully separate recurrent species effects from community turnover; inference should therefore remain at the community-wide species-by-plot level. Fifth, CUE was measured by an external analytical laboratory and the underlying growth, respiration and calculation metadata were not available; associations involving the microbial carbon axis should therefore be interpreted with this methodological limitation in mind.

Despite these limitations, our findings provide a coherent framework for understanding how herbaceous plants persist along dryland moisture gradients. Rather than shifting along a single synchronized whole-plant economic axis, dryland herbs adjusted selected leaf and root modules. Leaf structural protection and high specific root length were associated with drier conditions, whereas lateral-root proliferation increased towards wetter conditions. Leaf size was coordinated with root structural traits but diverged from specific root length, indicating that hydraulic or structural support and absorptive efficiency represent different below-ground pathways. Environmental models further showed that this coordination was associated with climate, local soil water, microbial carbon processing, SOC–N status, elevation and life form. Together, these results suggest that predicting plant functional responses to intensifying drought will require moving beyond single-organ or single-axis trait frameworks and incorporating the modular coordination of leaf and root functions in their local soil and landscape context.

## 5 Conclusions

Our study shows that dryland herbaceous plants along the Xinjiang aridity gradient did not respond through a uniform shift of the whole leaf–root phenotype. Instead, drought-related variation was concentrated in a small set of traits: thicker leaves and higher specific root length characterized the drier end of the gradient, whereas stronger lateral-root proliferation occurred under wetter conditions. Leaf–root coordination was therefore partial and trait-specific, with leaf size traits coupled to root length and diameter but opposed to specific root length. Environmental models further indicated that this coordination was associated not with aridity alone, but with the combined variation in soil water availability, microbial carbon processes, SOC–N nutrient status, elevation and life form. These findings suggest that dryland herbs maintain function through coupled but modular adjustments of leaf structure, absorptive root morphology and local root proliferation. Incorporating these below-ground and coordination dimensions should improve predictions of plant functional responses to increasing drought in water-limited ecosystems.

## Data Availability

The original contributions presented in the study are included in the article/**Supplementary Material**. Further inquiries can be directed to the corresponding author.

## Supplementary material

Supplementary Figure 1 Data structure, variable availability and life-form composition. **(a)**, Data hierarchy from raw field records to the final matched analytical dataset. The original dataset contained 629 field records, 87 named plots and 88 coordinate positions; the final dataset contained 474 matched species-level observations. **(b)**, Non-missing records for environmental, leaf and root variables before final matching. The dashed line indicates the final matched dataset size. **(c)**, Distribution of species-level observations along the aridity index gradient. **(d)**, Life-form composition of the final matched dataset.

Supplementary Figure 2 Life-form sensitivity of trait responses to aridity. Standardized effects of aridity index (AI) on leaf and root traits in the main annual and perennial herbaceous subsets. **(a)**, Leaf traits of annual species. **(b)**, Leaf traits of perennial species. **(c)**, Root traits of annual species. **(d)**, Root traits of perennial species. Points indicate standardized regression coefficients and horizontal bars indicate 95% confidence intervals. Asterisks indicate statistical significance. Sample sizes are shown in each panel and reflect the observations available for the corresponding model.

Supplementary Figure 3 Collinearity screening and construction of environmental composite axes. **(a)**, Spearman correlation matrix among candidate environmental predictors. **(b)**, Variance inflation factors before variable reduction. Dashed lines indicate VIF thresholds of 5 and 10. **(c)**, Variance inflation factors after variable reduction. **(d)**, PCA loadings used to construct composite environmental axes. The microbial carbon axis was derived from CUE and microbial biomass carbon, and the SOC–N nutrient axis was derived mainly from soil organic carbon and total nitrogen, with total phosphorus retained in the PCA but contributing weakly to PC1.

Supplementary Figure 4 Model diagnostics. Diagnostic plots for representative trait–aridity models. **(a)**, Standardized residuals versus fitted values. **(b)**, Normal Q–Q plots of residuals. **(c)**, Distributions of site-level random effects. **(d)**, Cook’s distance for influence diagnostics. These diagnostics were used to evaluate residual structure, approximate normality, random-effect behavior and influential observations.
