# Supplementary material for: Dryland herbs coordinate leaf protection, absorptive root morphology and lateral-root proliferation along aridity gradients
Source: Front Plant Sci. 2026 Aug 20;17:1926524. doi: 10.3389/fpls.2026.1926524 (PMC13538827; doi:10.3389/fpls.2026.1926524)
Supplement: Supplementary file 1 [file Image1.pdf]

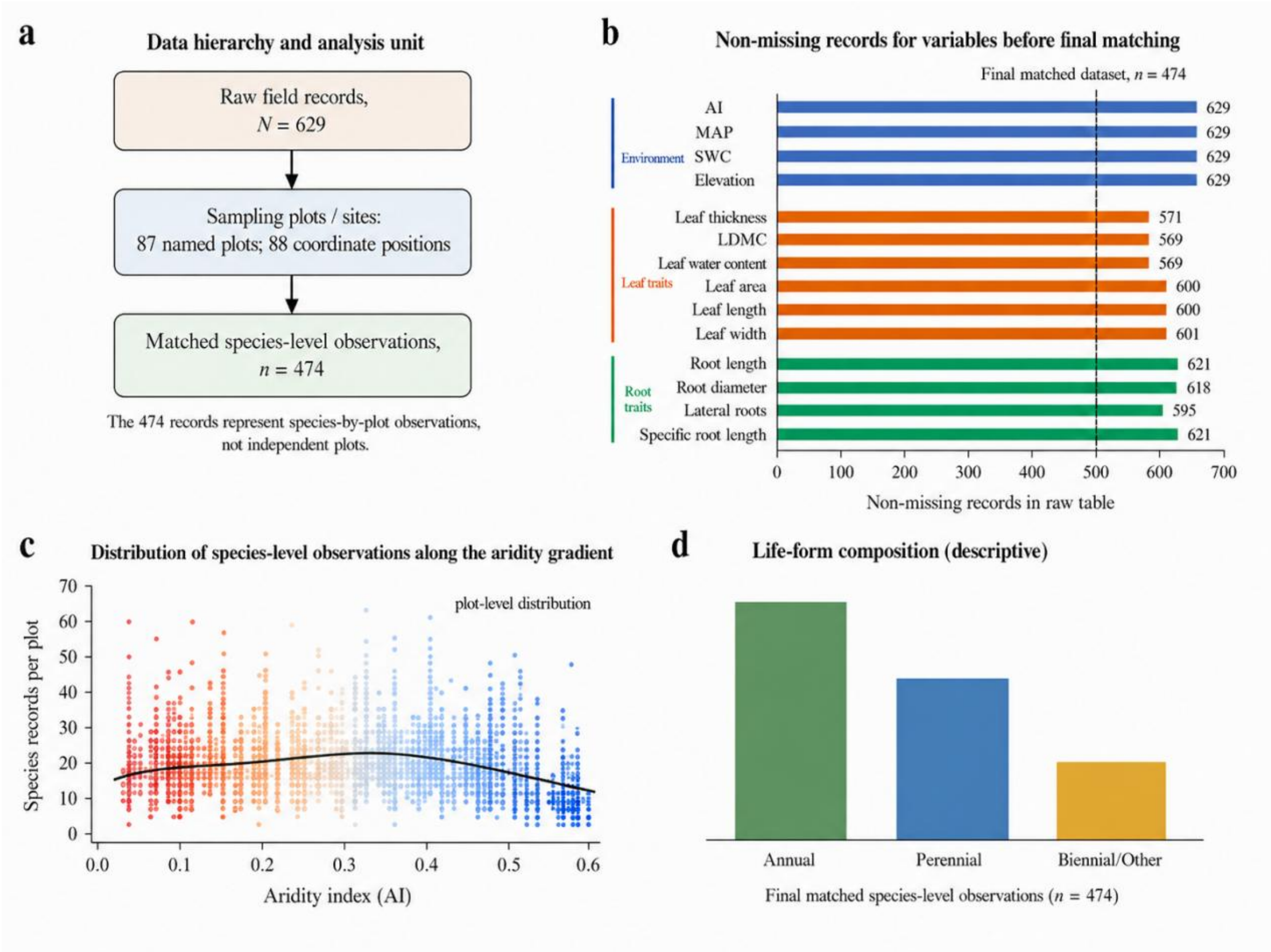

Figure 1 | Data structure, variable availability and life-form composition.

a, Data hierarchy from raw field records to the final matched analytical dataset. The original dataset contained 629 field records, 87 named plots and 88 coordinate positions; the final dataset contained 474 matched species-level observations. b, Non-missing records for environmental, leaf and root variables before final matching. The dashed line indicates the final matched dataset size. c, Distribution of species-level observations along the aridity index gradient. d, Life-form composition of the final matched dataset.
