# Supplementary material for: Dryland herbs coordinate leaf protection, absorptive root morphology and lateral-root proliferation along aridity gradients
Source: Front Plant Sci. 2026 Aug 20;17:1926524. doi: 10.3389/fpls.2026.1926524 (PMC13538827; doi:10.3389/fpls.2026.1926524)
Supplement: Supplementary file 2 [file Image2.pdf]

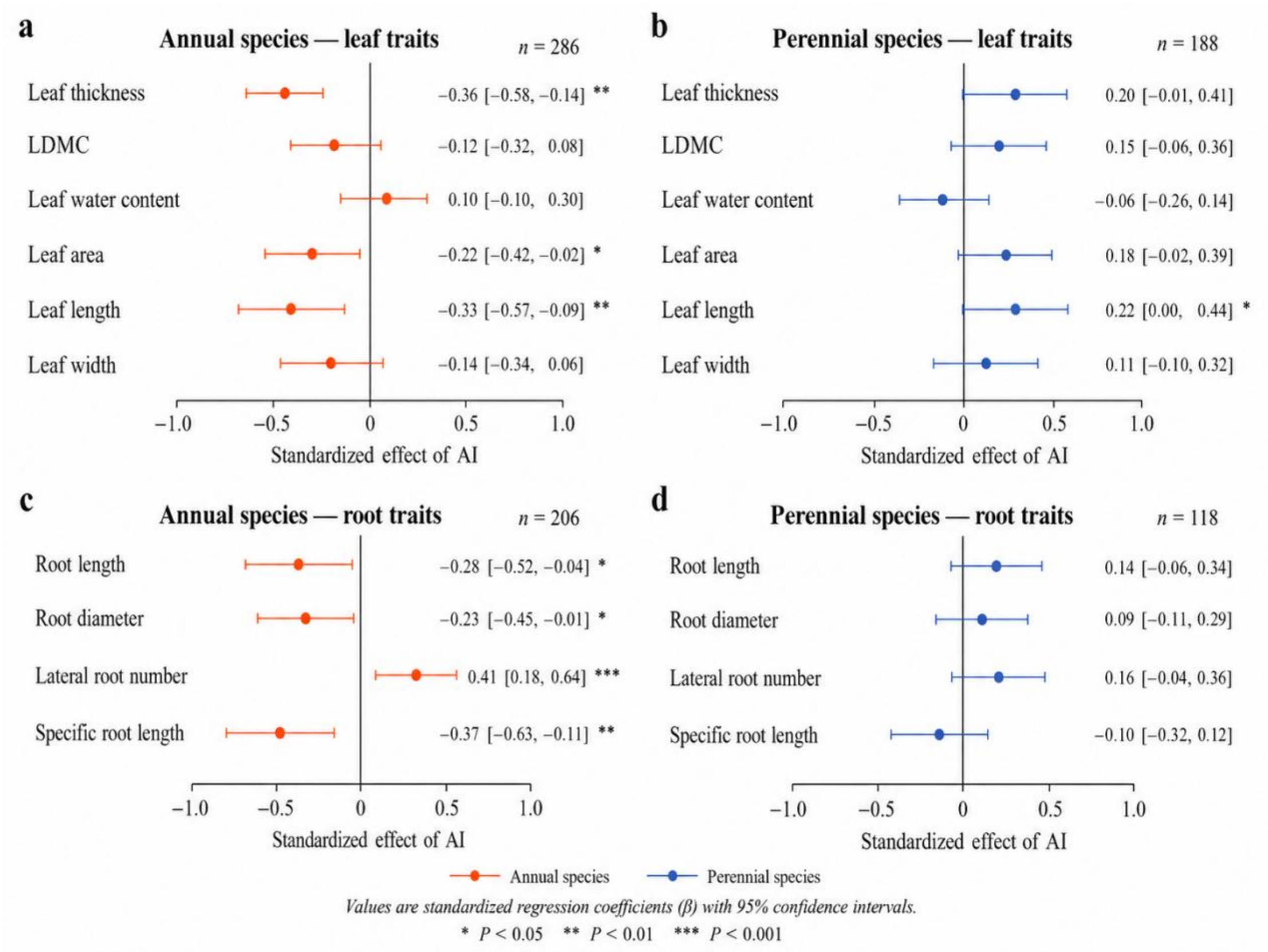


**Figure 2 | Life-form sensitivity of trait responses to aridity.**

Standardized effects of aridity index (AI) on leaf and root traits in the main annual and perennial herbaceous subsets. a, Leaf traits of annual species. b, Leaf traits of perennial species. c, Root traits of annual species. d, Root traits of perennial species. Points indicate standardized regression coefficients and horizontal bars indicate 95% confidence intervals. Asterisks indicate statistical significance. Sample sizes are shown in each panel and reflect the observations available for the corresponding model.
