# Supplementary material for: Dryland herbs coordinate leaf protection, absorptive root morphology and lateral-root proliferation along aridity gradients
Source: Front Plant Sci. 2026 Aug 20;17:1926524. doi: 10.3389/fpls.2026.1926524 (PMC13538827; doi:10.3389/fpls.2026.1926524)
Supplement: Supplementary file 3 [file Image3.pdf]

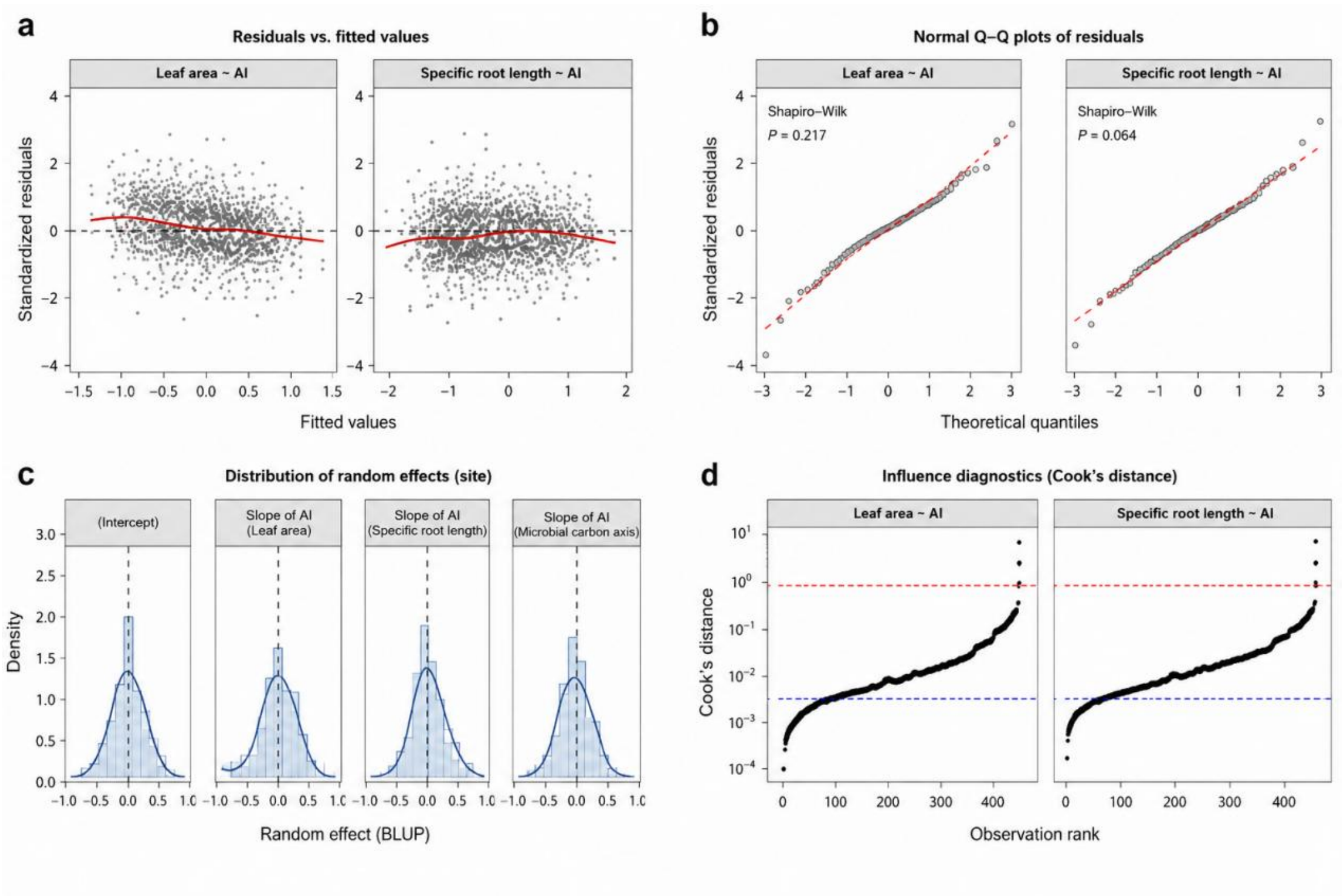

Figure 3 | Collinearity screening and construction of environmental composite axes.

a, Spearman correlation matrix among candidate environmental predictors. b, Variance inflation factors before variable reduction. Dashed lines indicate VIF thresholds of 5 and 10. c, Variance inflation factors after variable reduction. d, PCA loadings used to construct composite environmental axes. The microbial carbon axis was derived from CUE and microbial biomass carbon, and the SOC–N nutrient axis was derived mainly from soil organic carbon and total nitrogen, with total phosphorus retained in the PCA but contributing weakly to PC1.
