# Supplementary material for: Dryland herbs coordinate leaf protection, absorptive root morphology and lateral-root proliferation along aridity gradients
Source: Front Plant Sci. 2026 Aug 20;17:1926524. doi: 10.3389/fpls.2026.1926524 (PMC13538827; doi:10.3389/fpls.2026.1926524)
Supplement: Supplementary file 4 [file Image4.pdf]

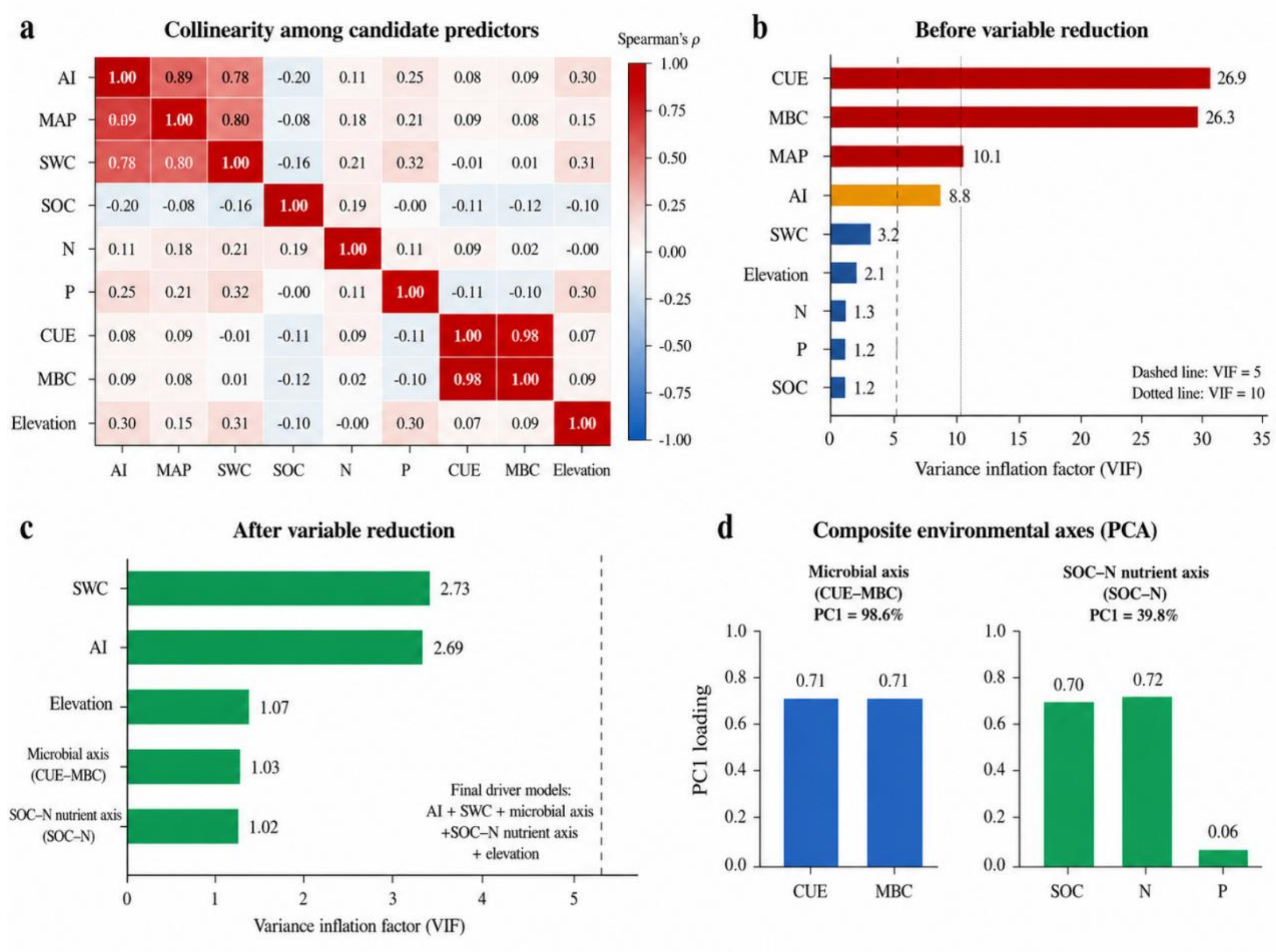

Figure 4 | Model diagnostics.

Diagnostic plots for representative trait–aridity models. a, Standardized residuals versus fitted values. b, Normal Q–Q plots of residuals. c, Distributions of site-level random effects. d, Cook's distance for influence diagnostics. These diagnostics were used to evaluate residual structure, approximate normality, random-effect behaviour and influential observations.
